# Bis­{μ-3,3′-(1,3,4-thia­diazole-2,5-diyl­dithio)bis­[penta­ne­dionato(1−)]}bis­[diaqua­nickel(II)] dimethyl­formamide disolvate trihydrate

**DOI:** 10.1107/S1600536808043699

**Published:** 2009-01-17

**Authors:** Fang-Fang Jian, Song-Jiang Han

**Affiliations:** a New Materials and Function Coordination Chemistry Laboratory, Qingdao University of Science and Technology, Qingdao 266042, People’s Republic of China

## Abstract

The title compound, [Ni_2_(C_12_H_12_N_2_O_4_S_3_)(H_2_O)_4_]·2C_3_H_7_NO·3H_2_O, is made up of a centrosymmetric, bimetallic complex containing a 24-membered macrocyclic ring, two dimethyl­formamide and three water solvent mol­ecules. The Ni atom adopts a slightly distorted NiO_6_ octahedral geometry arising from two *O*,*O*-bidentate ligands and two water molecules. There are inter­molecular O—H⋯O and O—H⋯N inter­actions in the crystal structure. One of the uncoordinated water molecules is diordered over two sets of sites of equal occupancy.

## Related literature

For background to metallamacrocycles, see: Gaynor *et al.* (2002 ▶); Shan *et al.* (2004 ▶); Weng *et al.* (2004 ▶); Zhang *et al.* (2006 ▶).
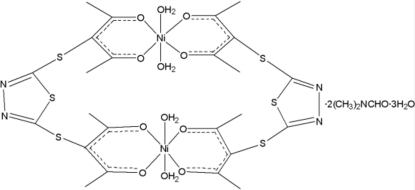


## Experimental

### 

#### Crystal data

[Ni_2_(C_12_H_12_N_2_O_4_S_3_)(H_2_O)_4_]·2C_3_H_7_NO·3H_2_O
*M* *_r_* = 1078.56
Triclinic, 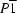

*a* = 10.582 (2) Å
*b* = 11.469 (1) Å
*c* = 12.136 (2) Å
α = 102.30 (3)°
β = 107.21 (1)°
γ = 116.26 (3)°
*V* = 1155.6 (6) Å^3^
*Z* = 1
Mo *K*α radiation
μ = 1.16 mm^−1^
*T* = 295 (2) K
0.23 × 0.20 × 0.16 mm

#### Data collection

Enraf–Nonius CAD-4 diffractometer
Absorption correction: none
6130 measured reflections
4039 independent reflections
3498 reflections with *I* > 2σ(*I*)
*R*_int_ = 0.013
3 standard reflections every 100 reflections intensity decay: none

#### Refinement

*R*[*F* ^2^ > 2σ(*F* ^2^)] = 0.035
*wR*(*F* ^2^) = 0.095
*S* = 1.03
4039 reflections
284 parameters
H-atom parameters constrained
Δρ_max_ = 0.47 e Å^−3^
Δρ_min_ = −0.60 e Å^−3^

### 

Data collection: *CAD-4 Software* (Enraf–Nonius, 1989 ▶); cell refinement: *CAD-4 Software*; data reduction: *CAD-4 Software*; program(s) used to solve structure: *SHELXS97* (Sheldrick, 2008 ▶); program(s) used to refine structure: *SHELXL97* (Sheldrick, 2008 ▶); molecular graphics: *SHELXTL/PC* (Sheldrick, 2008 ▶); software used to prepare material for publication: *WinGX* (Farrugia, 1999 ▶).

## Supplementary Material

Crystal structure: contains datablocks global, I. DOI: 10.1107/S1600536808043699/at2698sup1.cif

Structure factors: contains datablocks I. DOI: 10.1107/S1600536808043699/at2698Isup2.hkl

Additional supplementary materials:  crystallographic information; 3D view; checkCIF report

## Figures and Tables

**Table 1:** Selected bond lengths (Å)

Ni1—O4^i^	1.9851 (19)
Ni1—O2	1.992 (2)
Ni1—O3^i^	1.998 (2)
Ni1—O1	2.0029 (19)
Ni1—O2*W*	2.060 (2)
Ni1—O1*W*	2.065 (2)

Symmetry code: (i) 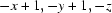.

**Table 2:** Hydrogen-bond geometry (Å, °)

*D*—H⋯*A*	*D*—H	H⋯*A*	*D*⋯*A*	*D*—H⋯*A*
O1*W*—H1*W*1⋯O3^ii^	0.85	2.48	2.817 (5)	104
O1*W*—H1*W*1⋯O1^iii^	0.85	2.11	2.932 (2)	162
O3*W*—H1*W*3⋯N2^iv^	0.85	2.04	2.865 (6)	164
O2*W*—H2*W*2⋯O5^i^	0.85	1.87	2.700 (4)	164

Symmetry codes: (i) 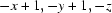; (ii) 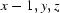; (iii) 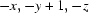; (iv) 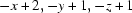.

**Fig. 1.**
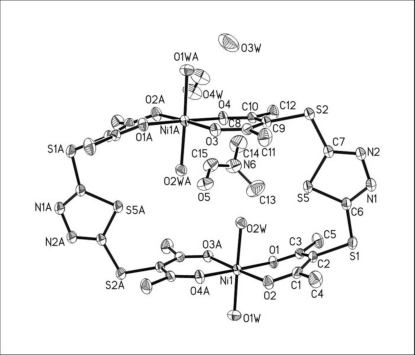
The molecular structure of the title compound with the atom-labeling scheme. Displacement ellipsoids are drawn at the 30% probability level.

**Fig. 2.**
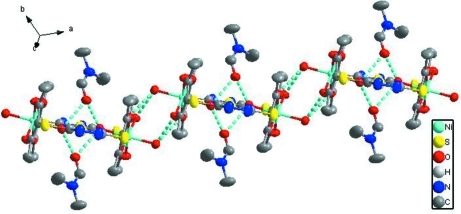
A view of the one-dimensional infinite hydrogen bonds chain. The green lines for hydrogen bonds in the plane, the hydrogen atoms are omited for clarity, the same hereafter.

**Fig. 3.**
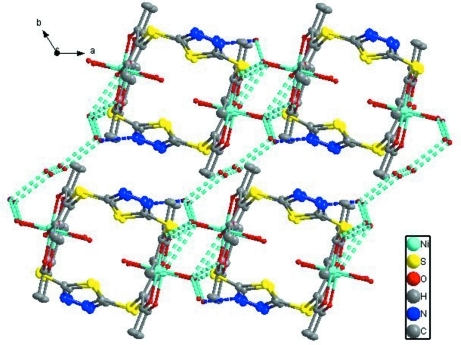
The three-dimensional network structures formed via hydrogen bonds along a-directions. The blue lines for O3W—H1W3..N2, For clarity, the solvent molecules of DMF and H atoms are not shown.

## supplementary crystallographic information

### Comment

The design and study of various metal-containing macrocycles and cages is one of the most active and interesting areas in modern supramolecular chemistry. The appealing structures of the metallamacrocycles can act as highly specific hosts used for selective recognition of neutral molecules, cations and anions. and switchable electron-transfer (Shan *et al.*, 2004), catalysis (Weng *et al.*, 2004) and magnetism (Gaynor *et al.*, 2002) have also been reported as the possible applications. Among the metallamacrocyclic complexes, dimetal macrocyclic complexes have attracted great interest in recent years and become an important type of metallamacrocycles. Herein, the title compound has been synthesized and we report its crystal structure.

The crystal structure are composed of the dimetal macrocyclic complexe and the solvent molecules of DMF and water (Fig.1). Accompanying the formation of the C—S bond, nickel acetyl acetonate [Ni(acac)_2_] and 2,5-dimercapto-1,3,4-thiadiazole (DMTD) construct a 24-membered dimetal macrocyclic complex. The symmetrical center exists within the dimetal macrocyclic structure. The Ni(II) ion is chelated by the acetyl acetonate (acac) groups forming two non-planar six-membered chelating rings. The acac oxygen atoms which occupied the equatorial plane of the octahedral metal with rather small dihedral angles between the O1—Ni—O2 and O3—Ni—O4 planes is 2.4 (1)°, which is similar to those reported previously (Zhang *et al.*, 2006). The dihedral angles between the thiadiazole ring and the acac-S planes is 85.9 (2)°. Two solvent ligands of water molecules are located at opposed positions with one extending to the inner space of the macrocycle, the plane which they located are nearly perpendicular to the plane of Ni(acac)_2_.

The intermolecular hydrogen bonds of O—H..O and O—H..N are present in the crystal structure. Two solvent molecules of DMF connect to the 24-membered macrocycle structure *via* two hydrogen bonds of O2W—H2W2···O5. The one-dimensional infinite chains formed through the hydrogen bonds of O1W—H1W1···O1 and O1W—H1W1..O3 (Fig.2.). The two-dimensional sheets constructed through the H-bonding interactions of O1W-H2W1..O3W and O4W—H1W4···O3W. Then, the upper N2 atoms of DMTD and the lower O3W atoms of water connect together to form the three-dimensional network structure *via* the hydrogen bond of O3W—H1W3..N2 (Fig. 3).

### Experimental

A mixture of DMTD (1.5 g, 0.01 mol) and sodium ethoxide (1.36 g,0.02 mol) was stirred with ethanol (50 mL) at room temperature for 30 min, then nickel acetyl acetonate (2.57 g, 0.01 mol) were added and heated to reflux about 4 h, yielded sky-blue precipitate, affording the title compound (3.34 g, yield 31%).Single crystals suitable for X-ray measurements were obtained by recrystallization from the mixture solvent of water and DMF (the mol ratio 1:5) at room temperature.

### Refinement

H atoms were positioned geometrically and allowed to ride on their parent atoms, with O—H and C—H distances of 0.85 and 0.96 Å, respectively, andwith *U*_iso_(H) = 1.2 or 1.5*U*_eq_ of the parent atoms.

### Crystal data

**Table:**

[Ni_2_(C_12_H_12_N_2_O_4_S_3_)(H_2_O)_4_]·2C_3_H_7_NO·3H_2_O	*Z* = 1
*M**_r_* = 1078.56	*F*(000) = 562
Triclinic, *P*1	*D*_x_ = 1.550 Mg m^−^^3^
Hall symbol: -P 1	Melting point: 554.6 K
*a* = 10.582 (2) Å	Mo *K*α radiation, λ = 0.71073 Å
*b* = 11.469 (1) Å	Cell parameters from 25 reflections
*c* = 12.136 (2) Å	θ = 4–14°
α = 102.30 (3)°	µ = 1.16 mm^−^^1^
β = 107.21 (1)°	*T* = 295 K
γ = 116.26 (3)°	Block, green
*V* = 1155.6 (6) Å^3^	0.23 × 0.20 × 0.16 mm

### Data collection

**Table:**

Enraf–Nonius CAD-4 diffractometer	*R*_int_ = 0.013
Radiation source: fine-focus sealed tube	θ_max_ = 25.0°, θ_min_ = 1.9°
graphite	*h* = −11→12
ω scans	*k* = −13→13
6130 measured reflections	*l* = −14→10
4039 independent reflections	3 standard reflections every 100 reflections
3498 reflections with *I* > 2σ(*I*)	intensity decay: none

### Refinement

**Table:**

Refinement on *F*^2^	Primary atom site location: structure-invariant direct methods
Least-squares matrix: full	Secondary atom site location: difference Fourier map
*R*[*F*^2^ > 2σ(*F*^2^)] = 0.035	Hydrogen site location: inferred from neighbouring sites
*wR*(*F*^2^) = 0.095	H-atom parameters constrained
*S* = 1.03	*w* = 1/[σ^2^(*F*_o_^2^) + (0.0451*P*)^2^ + 0.9813*P*] where *P* = (*F*_o_^2^ + 2*F*_c_^2^)/3
4039 reflections	(Δ/σ)_max_ < 0.001
284 parameters	Δρ_max_ = 0.47 e Å^−^^3^
0 restraints	Δρ_min_ = −0.60 e Å^−^^3^

### Special details

**Table:**

Geometry. All e.s.d.'s (except the e.s.d. in the dihedral angle between two l.s. planes) are estimated using the full covariance matrix. The cell e.s.d.'s are taken into account individually in the estimation of e.s.d.'s in distances, angles and torsion angles; correlations between e.s.d.'s in cell parameters are only used when they are defined by crystal symmetry. An approximate (isotropic) treatment of cell e.s.d.'s is used for estimating e.s.d.'s involving l.s. planes.
Refinement. Refinement of *F*^2^ against ALL reflections. The weighted *R*-factor *wR* and goodness of fit *S* are based on *F*^2^, conventional *R*-factors *R* are based on *F*, with *F* set to zero for negative *F*^2^. The threshold expression of *F*^2^ > σ(*F*^2^) is used only for calculating *R*-factors(gt) *etc*. and is not relevant to the choice of reflections for refinement. *R*-factors based on *F*^2^ are statistically about twice as large as those based on *F*, and *R*- factors based on ALL data will be even larger.

### Fractional atomic coordinates and isotropic or equivalent isotropic displacement parameters (Å^2^)

**Table:**

	*x*	*y*	*z*	*U*_iso_*/*U*_eq_	Occ. (<1)
Ni1	0.26742 (4)	0.62302 (4)	0.03339 (3)	0.03044 (12)	
S1	0.57596 (10)	0.85228 (10)	0.50283 (7)	0.0503 (2)	
S2	0.96862 (10)	0.64320 (10)	0.43743 (7)	0.0517 (2)	
S5	0.70843 (10)	0.70555 (9)	0.37703 (7)	0.0483 (2)	
O1	0.2162 (2)	0.5835 (2)	0.17277 (17)	0.0363 (4)	
O2	0.4302 (3)	0.8227 (2)	0.15619 (19)	0.0444 (5)	
O1W	0.1000 (3)	0.6732 (2)	−0.0185 (2)	0.0483 (5)	
H1W1	0.0024	0.6117	−0.0648	0.058*	
H2W1	0.1129	0.7547	−0.0008	0.058*	
O3	0.8900 (2)	0.5791 (2)	0.08790 (18)	0.0372 (4)	
O2W	0.4322 (2)	0.5712 (2)	0.08969 (19)	0.0413 (5)	
H1W2	0.4835	0.5644	0.1540	0.050*	
H2W2	0.4945	0.6091	0.0590	0.050*	
O4	0.6766 (2)	0.3368 (2)	0.10243 (18)	0.0401 (5)	
N1	0.8243 (3)	0.8407 (3)	0.6137 (2)	0.0542 (7)	
N2	0.9174 (3)	0.7907 (3)	0.5975 (2)	0.0529 (7)	
C1	0.5014 (4)	0.8636 (3)	0.2726 (3)	0.0396 (7)	
C2	0.4545 (3)	0.7796 (3)	0.3404 (3)	0.0375 (6)	
C3	0.3124 (3)	0.6462 (3)	0.2874 (3)	0.0359 (6)	
C4	0.6446 (5)	1.0111 (4)	0.3393 (4)	0.0639 (10)	
H4A	0.6582	1.0525	0.2795	0.096*	
H4B	0.7353	1.0081	0.3800	0.096*	
H4C	0.6324	1.0672	0.4012	0.096*	
C5	0.2640 (4)	0.5707 (4)	0.3676 (3)	0.0580 (9)	
H5A	0.1646	0.4824	0.3160	0.087*	
H5B	0.2543	0.6286	0.4303	0.087*	
H5C	0.3419	0.5524	0.4081	0.087*	
C6	0.7117 (4)	0.8037 (3)	0.5087 (3)	0.0412 (7)	
C7	0.8711 (4)	0.7194 (3)	0.4804 (3)	0.0410 (7)	
C8	0.9326 (3)	0.6343 (3)	0.2039 (3)	0.0370 (6)	
C9	0.8698 (3)	0.5594 (3)	0.2722 (3)	0.0376 (6)	
C10	0.7436 (3)	0.4150 (3)	0.2166 (3)	0.0364 (6)	
C11	1.0570 (4)	0.7901 (3)	0.2674 (3)	0.0622 (10)	
H11A	1.0861	0.8227	0.2069	0.093*	
H11B	1.1477	0.8063	0.3335	0.093*	
H11C	1.0169	0.8410	0.3025	0.093*	
C12	0.6796 (5)	0.3461 (4)	0.2957 (3)	0.0558 (9)	
H12A	0.5956	0.2490	0.2436	0.084*	
H12B	0.6404	0.3956	0.3341	0.084*	
H12C	0.7617	0.3495	0.3600	0.084*	
O5	0.3257 (3)	0.2918 (3)	−0.0367 (3)	0.0627 (7)	
N6	0.2517 (4)	0.1686 (4)	0.0772 (4)	0.0702 (9)	
C13	0.2358 (8)	0.2730 (6)	0.1515 (6)	0.110 (2)	
H13A	0.2517	0.3453	0.1194	0.165*	
H13B	0.3128	0.3150	0.2376	0.165*	
H13C	0.1322	0.2280	0.1469	0.165*	
C14	0.2237 (8)	0.0463 (5)	0.1088 (6)	0.1039 (18)	
H14A	0.2292	−0.0190	0.0491	0.156*	
H14B	0.1214	−0.0001	0.1063	0.156*	
H14C	0.3023	0.0774	0.1918	0.156*	
C15	0.2982 (5)	0.1897 (4)	−0.0082 (4)	0.0663 (11)	
H15A	0.2931	0.1006	−0.0592	0.079*	
O3W	0.8641 (5)	0.1907 (5)	0.1821 (4)	0.1309 (16)	
H1W3	0.9173	0.1787	0.2412	0.157*	
H2W3	0.8417	0.2476	0.2148	0.157*	
O4W	0.4494 (6)	−0.0168 (7)	−0.0250 (7)	0.089 (2)	0.50
H1W4	0.3783	−0.0739	−0.0101	0.107*	0.50
H2W4	0.4044	−0.0037	−0.0875	0.107*	0.50

### Atomic displacement parameters (Å^2^)

**Table:**

	*U*^11^	*U*^22^	*U*^33^	*U*^12^	*U*^13^	*U*^23^
Ni1	0.0302 (2)	0.0303 (2)	0.02522 (19)	0.01252 (15)	0.01182 (15)	0.01108 (14)
S1	0.0508 (5)	0.0655 (5)	0.0274 (4)	0.0360 (4)	0.0116 (3)	0.0044 (4)
S2	0.0520 (5)	0.0692 (6)	0.0281 (4)	0.0411 (5)	0.0063 (3)	0.0073 (4)
S5	0.0499 (5)	0.0640 (5)	0.0237 (4)	0.0367 (4)	0.0069 (3)	0.0057 (3)
O1	0.0323 (10)	0.0410 (11)	0.0295 (10)	0.0144 (9)	0.0148 (8)	0.0145 (9)
O2	0.0502 (13)	0.0328 (11)	0.0331 (11)	0.0141 (10)	0.0126 (10)	0.0118 (9)
O1W	0.0495 (13)	0.0527 (13)	0.0606 (14)	0.0344 (11)	0.0284 (11)	0.0324 (11)
O3	0.0346 (10)	0.0328 (10)	0.0341 (11)	0.0126 (9)	0.0138 (9)	0.0116 (9)
O2W	0.0397 (11)	0.0540 (13)	0.0381 (11)	0.0274 (10)	0.0205 (9)	0.0226 (10)
O4	0.0414 (11)	0.0387 (11)	0.0293 (11)	0.0135 (9)	0.0155 (9)	0.0138 (9)
N1	0.0613 (18)	0.0711 (19)	0.0285 (14)	0.0439 (16)	0.0124 (13)	0.0096 (13)
N2	0.0566 (17)	0.0703 (19)	0.0276 (13)	0.0420 (16)	0.0089 (12)	0.0090 (13)
C1	0.0405 (16)	0.0349 (15)	0.0353 (16)	0.0201 (13)	0.0132 (13)	0.0062 (13)
C2	0.0397 (16)	0.0431 (16)	0.0258 (14)	0.0239 (14)	0.0119 (12)	0.0077 (12)
C3	0.0388 (16)	0.0457 (16)	0.0306 (15)	0.0266 (14)	0.0181 (13)	0.0155 (13)
C4	0.061 (2)	0.0381 (18)	0.053 (2)	0.0113 (17)	0.0108 (18)	0.0078 (16)
C5	0.059 (2)	0.071 (2)	0.0419 (19)	0.0280 (19)	0.0273 (17)	0.0299 (18)
C6	0.0438 (17)	0.0480 (18)	0.0274 (15)	0.0252 (15)	0.0131 (13)	0.0105 (13)
C7	0.0420 (16)	0.0460 (17)	0.0269 (15)	0.0235 (14)	0.0093 (13)	0.0100 (13)
C8	0.0336 (15)	0.0366 (15)	0.0350 (16)	0.0193 (13)	0.0112 (12)	0.0098 (13)
C9	0.0381 (16)	0.0434 (16)	0.0263 (14)	0.0242 (14)	0.0090 (12)	0.0085 (12)
C10	0.0410 (16)	0.0472 (17)	0.0290 (15)	0.0275 (14)	0.0164 (13)	0.0192 (13)
C11	0.061 (2)	0.0355 (18)	0.052 (2)	0.0108 (17)	0.0151 (18)	0.0028 (15)
C12	0.071 (2)	0.062 (2)	0.0417 (18)	0.0329 (19)	0.0325 (18)	0.0279 (17)
O5	0.0641 (16)	0.0535 (15)	0.095 (2)	0.0349 (13)	0.0507 (15)	0.0428 (14)
N6	0.076 (2)	0.062 (2)	0.094 (3)	0.0371 (18)	0.052 (2)	0.0472 (19)
C13	0.160 (6)	0.112 (4)	0.141 (5)	0.095 (4)	0.115 (5)	0.077 (4)
C14	0.151 (5)	0.073 (3)	0.122 (4)	0.061 (3)	0.082 (4)	0.064 (3)
C15	0.071 (3)	0.052 (2)	0.087 (3)	0.033 (2)	0.045 (2)	0.031 (2)
O3W	0.143 (4)	0.201 (5)	0.117 (3)	0.117 (4)	0.066 (3)	0.113 (3)
O4W	0.063 (5)	0.068 (4)	0.109 (6)	0.023 (5)	0.018 (4)	0.046 (4)

### Geometric parameters (Å, °)

**Table:**

Ni1—O4^i^	1.9851 (19)	C4—H4C	0.9600
Ni1—O2	1.992 (2)	C5—H5A	0.9600
Ni1—O3^i^	1.998 (2)	C5—H5B	0.9600
Ni1—O1	2.0029 (19)	C5—H5C	0.9600
Ni1—O2W	2.060 (2)	C8—C9	1.411 (4)
Ni1—O1W	2.065 (2)	C8—C11	1.500 (4)
S1—C6	1.745 (3)	C9—C10	1.419 (4)
S1—C2	1.756 (3)	C10—C12	1.496 (4)
S2—C7	1.739 (3)	C11—H11A	0.9600
S2—C9	1.757 (3)	C11—H11B	0.9600
S5—C7	1.720 (3)	C11—H11C	0.9600
S5—C6	1.728 (3)	C12—H12A	0.9600
O1—C3	1.256 (3)	C12—H12B	0.9600
O2—C1	1.248 (4)	C12—H12C	0.9600
O1W—H1W1	0.8500	O5—C15	1.228 (4)
O1W—H2W1	0.8500	N6—C15	1.295 (5)
O3—C8	1.256 (3)	N6—C13	1.443 (6)
O3—Ni1^i^	1.998 (2)	N6—C14	1.460 (5)
O2W—H1W2	0.8500	C13—H13A	0.9600
O2W—H2W2	0.8501	C13—H13B	0.9600
O4—C10	1.247 (3)	C13—H13C	0.9600
O4—Ni1^i^	1.9851 (19)	C14—H14A	0.9600
N1—C6	1.284 (4)	C14—H14B	0.9600
N1—N2	1.381 (4)	C14—H14C	0.9600
N2—C7	1.290 (4)	C15—H15A	1.0463
C1—C2	1.418 (4)	O3W—H1W3	0.8498
C1—C4	1.495 (5)	O3W—H2W3	0.8500
C2—C3	1.410 (4)	O4W—O4W^ii^	0.901 (10)
C3—C5	1.490 (4)	O4W—H1W4	0.8500
C4—H4A	0.9600	O4W—H2W4	0.8500
C4—H4B	0.9600		
			
O4^i^—Ni1—O2	91.40 (9)	H5B—C5—H5C	109.5
O4^i^—Ni1—O3^i^	88.63 (9)	N1—C6—S5	114.8 (2)
O2—Ni1—O3^i^	177.92 (9)	N1—C6—S1	121.4 (2)
O4^i^—Ni1—O1	178.59 (8)	S5—C6—S1	123.82 (17)
O2—Ni1—O1	87.98 (9)	N2—C7—S5	114.7 (2)
O3^i^—Ni1—O1	91.95 (9)	N2—C7—S2	120.5 (2)
O4^i^—Ni1—O2W	91.54 (9)	S5—C7—S2	124.88 (17)
O2—Ni1—O2W	88.80 (9)	O3—C8—C9	124.0 (3)
O3^i^—Ni1—O2W	89.12 (9)	O3—C8—C11	114.9 (3)
O1—Ni1—O2W	87.18 (8)	C9—C8—C11	121.0 (3)
O4^i^—Ni1—O1W	90.39 (9)	C8—C9—C10	124.1 (3)
O2—Ni1—O1W	91.24 (10)	C8—C9—S2	117.8 (2)
O3^i^—Ni1—O1W	90.85 (10)	C10—C9—S2	117.7 (2)
O1—Ni1—O1W	90.90 (9)	O4—C10—C9	124.8 (3)
O2W—Ni1—O1W	178.07 (8)	O4—C10—C12	114.8 (3)
C6—S1—C2	102.53 (14)	C9—C10—C12	120.4 (3)
C7—S2—C9	105.44 (14)	C8—C11—H11A	109.5
C7—S5—C6	86.11 (15)	C8—C11—H11B	109.5
C3—O1—Ni1	124.99 (18)	H11A—C11—H11B	109.5
C1—O2—Ni1	126.2 (2)	C8—C11—H11C	109.5
Ni1—O1W—H1W1	123.7	H11A—C11—H11C	109.5
Ni1—O1W—H2W1	128.6	H11B—C11—H11C	109.5
H1W1—O1W—H2W1	107.7	C10—C12—H12A	109.5
C8—O3—Ni1^i^	128.97 (19)	C10—C12—H12B	109.5
Ni1—O2W—H1W2	139.2	H12A—C12—H12B	109.5
Ni1—O2W—H2W2	104.0	C10—C12—H12C	109.5
H1W2—O2W—H2W2	107.7	H12A—C12—H12C	109.5
C10—O4—Ni1^i^	128.75 (19)	H12B—C12—H12C	109.5
C6—N1—N2	112.1 (3)	C15—N6—C13	120.0 (4)
C7—N2—N1	112.3 (3)	C15—N6—C14	122.7 (4)
O2—C1—C2	124.1 (3)	C13—N6—C14	117.2 (4)
O2—C1—C4	115.3 (3)	N6—C13—H13A	109.5
C2—C1—C4	120.6 (3)	N6—C13—H13B	109.5
C3—C2—C1	124.2 (3)	H13A—C13—H13B	109.5
C3—C2—S1	118.3 (2)	N6—C13—H13C	109.5
C1—C2—S1	117.4 (2)	H13A—C13—H13C	109.5
O1—C3—C2	124.1 (3)	H13B—C13—H13C	109.5
O1—C3—C5	114.9 (3)	N6—C14—H14A	109.5
C2—C3—C5	120.9 (3)	N6—C14—H14B	109.5
C1—C4—H4A	109.5	H14A—C14—H14B	109.5
C1—C4—H4B	109.5	N6—C14—H14C	109.5
H4A—C4—H4B	109.5	H14A—C14—H14C	109.5
C1—C4—H4C	109.5	H14B—C14—H14C	109.5
H4A—C4—H4C	109.5	O5—C15—N6	125.1 (4)
H4B—C4—H4C	109.5	O5—C15—H15A	123.6
C3—C5—H5A	109.5	N6—C15—H15A	110.6
C3—C5—H5B	109.5	H1W3—O3W—H2W3	107.7
H5A—C5—H5B	109.5	O4W^ii^—O4W—H1W4	118.3
C3—C5—H5C	109.5	O4W^ii^—O4W—H2W4	134.0
H5A—C5—H5C	109.5	H1W4—O4W—H2W4	107.7
			
O2—Ni1—O1—C3	−29.7 (2)	C7—S5—C6—S1	−179.8 (2)
O3^i^—Ni1—O1—C3	148.2 (2)	C2—S1—C6—N1	178.6 (3)
O2W—Ni1—O1—C3	59.2 (2)	C2—S1—C6—S5	−0.7 (3)
O1W—Ni1—O1—C3	−121.0 (2)	N1—N2—C7—S5	0.0 (4)
O4^i^—Ni1—O2—C1	−151.7 (3)	N1—N2—C7—S2	−178.8 (2)
O1—Ni1—O2—C1	27.1 (3)	C6—S5—C7—N2	−0.4 (3)
O2W—Ni1—O2—C1	−60.1 (3)	C6—S5—C7—S2	178.3 (2)
O1W—Ni1—O2—C1	117.9 (3)	C9—S2—C7—N2	−179.4 (3)
C6—N1—N2—C7	0.7 (4)	C9—S2—C7—S5	1.9 (3)
Ni1—O2—C1—C2	−14.5 (4)	Ni1^i^—O3—C8—C9	−1.8 (4)
Ni1—O2—C1—C4	165.2 (2)	Ni1^i^—O3—C8—C11	178.8 (2)
O2—C1—C2—C3	−7.7 (5)	O3—C8—C9—C10	−4.6 (5)
C4—C1—C2—C3	172.7 (3)	C11—C8—C9—C10	174.8 (3)
O2—C1—C2—S1	176.6 (2)	O3—C8—C9—S2	167.6 (2)
C4—C1—C2—S1	−3.0 (4)	C11—C8—C9—S2	−13.0 (4)
C6—S1—C2—C3	96.7 (2)	C7—S2—C9—C8	96.1 (3)
C6—S1—C2—C1	−87.3 (3)	C7—S2—C9—C10	−91.2 (3)
Ni1—O1—C3—C2	20.1 (4)	Ni1^i^—O4—C10—C9	6.6 (4)
Ni1—O1—C3—C5	−161.4 (2)	Ni1^i^—O4—C10—C12	−174.2 (2)
C1—C2—C3—O1	4.4 (5)	C8—C9—C10—O4	2.1 (5)
S1—C2—C3—O1	−179.9 (2)	S2—C9—C10—O4	−170.1 (2)
C1—C2—C3—C5	−173.9 (3)	C8—C9—C10—C12	−177.1 (3)
S1—C2—C3—C5	1.7 (4)	S2—C9—C10—C12	10.7 (4)
N2—N1—C6—S5	−1.1 (4)	C13—N6—C15—O5	−3.0 (7)
N2—N1—C6—S1	179.6 (2)	C14—N6—C15—O5	−178.7 (5)
C7—S5—C6—N1	0.9 (3)		

Symmetry codes: (i) −*x*+1, −*y*+1, −*z*; (ii) −*x*+1, −*y*, −*z*.

### Hydrogen-bond geometry (Å, °)

**Table:**

*D*—H···*A*	*D*—H	H···*A*	*D*···*A*	*D*—H···*A*
O1W—H1W1···O3^iii^	0.85	2.48	2.817 (5)	104
O1W—H1W1···O1^iv^	0.85	2.11	2.932 (2)	162
O3W—H1W3···N2^v^	0.85	2.04	2.865 (6)	164
O2W—H2W2···O5^i^	0.85	1.87	2.700 (4)	164

Symmetry codes: (iii) *x*−1, *y*, *z*; (iv) −*x*, −*y*+1, −*z*; (v) −*x*+2, −*y*+1, −*z*+1; (i) −*x*+1, −*y*+1, −*z*.
